# From Protecting the Heart to Improving Athletic Performance – the Benefits of Local and Remote Ischaemic Preconditioning

**DOI:** 10.1007/s10557-015-6621-6

**Published:** 2015-10-19

**Authors:** Vikram Sharma, Reuben Marsh, Brian Cunniffe, Marco Cardinale, Derek M. Yellon, Sean M. Davidson

**Affiliations:** Department of Internal Medicine, Cleveland Clinic, Cleveland, OH USA; The Hatter Cardiovascular Institute, University College London, 67 Chenies Mews, London, WC1E 6HX UK; English institute of Sport, Bisham, Marlow UK; Institute of Sport, Exercise and Health, UCL, London, UK; Aspire Academy, Doha, Qatar

**Keywords:** Remote ischemic preconditioning, Exercise performance, Sports, Cardioprotection, Ischemia-reperfusion injury, CABG, PCI, Perconditioning, Postconditioning, Acute kidney injury

## Abstract

Remote Ischemic Preconditioning (RIPC) is a non-invasive cardioprotective intervention that involves brief cycles of limb ischemia and reperfusion. This is typically delivered by inflating and deflating a blood pressure cuff on one or more limb(s) for several cycles, each inflation-deflation being 3–5 min in duration. RIPC has shown potential for protecting the heart and other organs from injury due to lethal ischemia and reperfusion injury, in a variety of clinical settings. The mechanisms underlying RIPC are under intense investigation but are just beginning to be deciphered. Emerging evidence suggests that RIPC has the potential to improve exercise performance, via both local and remote mechanisms. This review discusses the clinical studies that have investigated the role of RIPC in cardioprotection as well as those studying its applicability in improving athletic performance, while examining the potential mechanisms involved.

## Introduction

Ischaemic preconditioning (IPC) is a phenomenon in which transient episodes of ischemia and reperfusion administered to an organ attenuate the lethal cellular injury sustained from a subsequent, prolonged ischaemic insult of the same organ. IPC was first described in a study by Murray et al. in 1986 [1], in which, the hearts of anaesthetized dogs were preconditioned with four 5 min occlusions of the circumflex artery, each separated by 5 min of reperfusion. This was followed by a sustained 40 min occlusion and 4 days of reperfusion. The extent of myocardial infarction in the preconditioned hearts was found to be dramatically reduced to a mere 25 % of that seen in the control hearts which did not receive preconditioning [1]. Later, IPC was also shown to have the ability to prevent lethal ischemia and reperfusion injury in skeletal muscles, and to protect the endothelium [2, 3].

Subsequently, the intriguing observation was made that protection of the heart could also be achieved by applying cycles of brief ischemia, alternating with reperfusion, to a tissue or organ *remote* from the heart - a concept named remote ischaemic preconditioning (RIPC). A crucial intermediate step towards the discovery of RIPC was made by Przyklenk et al. [4] in 1993, who demonstrated that preconditioning the territory of the heart supplied by the circumflex coronary artery also reduced the size of the infarct arising from the subsequent occlusion of the left anterior descending coronary artery. They called this phenomenon “preconditioning at a distance” [4]. This was followed by studies showing that preconditioning of the heart could be achieved by applying the brief episodes of ischemia and reperfusion to a remote organ such as the kidney or other abdominal visceral organs [5, 6]. Birnbaum et al. made the critical observation that RIPC could also be applied to the limb. In their experiments, they combined brief cycles of blood flow restriction with electrical stimulation of the gastrocnemius muscle in the same limb in order to induce demand ischemia [7]. When applied prior to sustained coronary artery occlusion and reperfusion, this intervention reduced infarct size by more than 65 % [7]. Kharbanda et al. were the first to demonstrate that the application of an RIPC stimulus without the need for electrical stimulation, reduced the extent of myocardial infarction in-vivo in pigs, and also attenuated endothelial injury in humans [8]. This study paved the way for the clinical application of RIPC by recognising the possibility of a non-invasive method of protecting the heart against lethal IR injury. Other studies demonstrated that in addition to protecting the heart, limb RIPC can also protect other organs including the kidneys, lungs, brain, and liver [9], as well as the endothelium [10] from injury caused by sustained ischemia and reperfusion.

In addition to the benefits of IPC and RIPC on the heart and the endothelium, both in terms of increased resistance to ischaemic injury and preservation of function in the face of ischemia and reperfusion, it has been hypothesised that IPC applied to the limb may have the potential to improve exercise performance via both local effects (i.e.,: to the limb) and remote effects (via the cardiovascular or nervous system). We refer to this approach here as “limb IPC” to distinguish it from the concept of using RIPC to target the remote organ alone. This review will appraise and discuss the studies that have evaluated the role of RIPC in preventing myocardial IR injury, as well as discussing the potential local and remote effects of limb IPC in improving exercise performance.

## Protecting the Heart with Remote Ischaemic Preconditioning

### Clinical Applications

RIPC has been shown to be a promising technique for reducing ischaemic myocardial cell death in various animal studies [4–8]. Although the procedure has been successfully applied following myocardial infarction in proof-of concept clinical trials [11–16], its clinical application is more conveniently studied in settings in which a sustained ischaemic insult can be predicted, which allows it to be administered prior to the ischemic insult. For example, some elevation of cardiac enzymes typically occurs peri-operatively in patients undergoing coronary artery bypass grafting [17]. Myocardial infarction occurring in this setting is termed “type 5” myocardial infarction [17]. Cardiac surgery therefore is a controlled clinical setting amenable to the investigation of the cardioprotective effects RIPC (Table 1).

**Table 1:** Clinical trials exploring benefits of RIPC in patients undergoing coronary artery bypass grafting (I = Ischemia, R = reperfusion)

Study	Number of patients	RIPC stimulus: limb(s) used, duration of I-R (min), number of RIPC cycles, cuff inflation pressure	Outcome
Cheung et al. [18]	37	Lower limb 5′ I 5′ R (x4); 15 mmHg above systolic arterial BP	Reduced cTnT release, Reduced inotrope requirement after surgery, Reduced airway resistance
Venugopal et al. [26]	45	Upper limb 5′ I 5′ R (x3); 200 mmHg	Reduced absolute cTnT release post surgery
Hausenloy et al. [22]	57	Upper limb 5′ I 5′ R (x3); 200 mmHg	Reduced cTnT release postoperatively
Thielmann et al. [25]	53	Upper limb 5′ I 5′ R (x3); 200 mmHg	Reduced cTnI release postoperatively
Gunaydin et al. [21]	8	Lower limb 3′ I 2′ R (x2); 200 mmHg	No change in CK-MB
Rahman et al. [24]	162	Upper limb 5′ I 5′ R (x3); 200 mmHg	Troponin-T release unaffected by RIPC
Wagner et al. [27]	101	Upper limb 5′ I 5′ R (x3); 40 mm Hg above resting systolic BP- given 18 h prior to surgery	Reduced cTnI release post-operatively
Kottenberg et al. [23]	72	Upper limb 5′ I 5′ R (x3); 200 mmHg	RIPC during isoflurane anaesthesia reduces post-op cTnI release, but not during Propofol anesthesia
Candilio et al. [19]	180	One upper and one lower limb simultaneously 5′I 5′R (x2); 200 mm Hg or to 15 mmHg above systolic BP if systolic BP >185 mmHg	Reduced perioperative TnI release, reduced incidence of post-op AF/AKI and duration of ICU stay
Gedik et al. [20]	20	Upper limb 5′ I 5′ R (x3)	Reduced TnI release post-operatively
Hausenloy et al. [30]	1,610	Upper limb 5′ I 5′ R (x4); 200 mm Hg	No significant difference in 30 day MACCE or 1 year clinical outcomes

Cheung et al. [18] were the first to successfully use RIPC in patients undergoing cardiac surgery, in a study assessing the effects of RIPC on children undergoing surgery to repair congenital heart defects. RIPC was induced by four 5 min cycles of lower-limb ischemia and reperfusion by inflation of a blood pressure (BP) cuff to 15 mmHg above the resting systolic arterial pressure (measured invasively via an arterial line), and compared against a control group who received no RIPC. They uncovered multiple positive effects of RIPC administered prior to surgery. Postoperative levels of troponin I and postoperative inotropic requirement were reduced in the RIPC group indicating less myocardial injury and better recovery of contractile function. In addition, the RIPC group had significantly lower airway resistance postoperatively.

The effects of RIPC on patients undergoing coronary artery bypass surgery (CABG) have been widely explored since, but with inconsistent findings [18–27]. Table 1 lists studies exploring the ability of RIPC given prior to CABG to mitigate myocardial injury (measured by rise in cardiac enzymes post operatively) and improve clinical outcomes post CABG surgery. Results have been mixed, with some demonstrating that RIPC is beneficial, reducing the extent of cardiac enzyme release, and others reporting no additional benefit over standard therapies (Table 1). An important caveat with these studies is that it is unclear to what extent the reduction of cardiac enzymes post operatively can be expected to translate to an improvement in clinical outcomes.

Another limitation in the clinical application of RIPC is the lack of a standardized optimal protocol for delivery of the RIPC stimulus in humans. The currently used protocols have been extrapolated from animal studies and there is a lack of evidence to guide what dose of RIPC is sufficient to trigger a cardioprotective response. We have recently shown that the magnitude of ischemia that occurs in the limb during RIPC differs widely based on the limb (upper vs. lower) and the cuff inflation pressure used [28], suggesting a need for more research to standardise the RIPC protocol, so that it can be delivered in a consistent manner in humans. Table 1 emphasizes the broad variations in RIPC protocols that have been applied in clinical trials. Differences include: the number of cycles of RIPC (typically two to four), length of the cycles (3 to 5 min), the choice of limb(s) used to deliver RIPC, and cuff inflation pressure which may be either a predetermined pressure or a pre-defined level above the resting systolic blood pressure. These differences may account for some of the variation in results noted with RIPC in the setting of CABG. An additional factor that may influence the results of these clinical trials is the choice and timing of anaesthetic agents used during surgery, since it is known that certain volatile anaesthetic agents can themselves precondition the heart against ischemic insults [29].

Other confounding factors may interfere with RIPC in patients undergoing CABG, including age, co-morbidities and other medications that the patients are taking peri-operatively. In addition, the mechanisms of myocardial injury and cardiac enzyme elevation in this setting are fairly diverse, and include type 2 myocardial infarction, macrovascular events such as graft failure, injury to the myocardium from surgical manipulation, microvascular injury and release of a cytosolic pool of cardiac enzymes without necrosis [17]. These factors result in only a very small target potentially amenable to protection that may be overwhelmed by the contribution from other sources of cardiac injury, making the benefits of RIPC in this setting more difficult to ascertain.

A further hurdle is the current paucity of data regarding the optimal time window of protection from RIPC and the duration of its effect. The duration of CABG surgery itself can vary significantly making it difficult to accurately time RIPC intervention prior to CABG. Finally, the myocardium is already protected during CABG by the use of standard cardioplegia techniques, which themselves are extremely effective in minimizing ischemic insult to the myocardium.

Considering these challenges, it is perhaps not entirely surprising that the first large, double-blinded, randomized, multi-centre study of RIPC in 1,610 patients undergoing CABG (the ERICCA trial) [30], did not show a significant clinical improvement using a primary combined endpoint of cardiovascular death, non-fatal myocardial infarction, coronary revascularization and stroke after 1 year (results presented at ACC 64th annual scientific meeting) [30]. More unexpectedly, however, RIPC did not significantly reduce the release of biomarkers of cardiac injury. One interpretation of these results is that CABG may not, in fact, be the ideal experimental setting for application of RIPC, particularly in view of the factors mentioned above.

Importantly, in a multicentre, single-blinded, randomized controlled trial of 519 patients experiencing ST-segment elevation myocardial infarction who were administered thrombolysis in the developing nation of Mauritius, significantly less release of cardiac enzymes was measured in those who received RIPC [16]. This inspires confidence that, in a setting where the degree of cardiac injury due to the ischaemia and reperfusion injury is more marked, the benefit of RIPC can be clearly observed.

The cardio-protective effects of RIPC are also being explored in other clinical settings, such as in patients undergoing elective percutaneous coronary intervention (PCI). In this scenario, myocardial injury can be anticipated [17], but contrast-induced acute kidney injury (nephropathy) may also occur, and has been proposed as an additional target for protection by RIPC. The results of trials evaluating the benefits of RIPC in elective PCI are fairly mixed (Table 2), in terms of both the effect of RIPC on peri-procedural myocardial infarction and clinical outcomes following PCI [31–40].

**Table 2:** Clinical studies and meta-analyses investigating the benefits of RIPC/RIPostC in patients undergoing PCI in a variety of settings (elective/NSTEMI/STEMI)

Study	Number of patients	RIPC stimulus: Limb(s) used, duration of ischemic and reperfusion episodes in min (where specified), number of cycles, cuff inflation pressure, clinical setting	Timing of RIPC in relation to PCI	Outcome
RIPC in non-emergent PCI				
Pei et al. (Metanalysis) [42]	1,713	Upper/Lower limb 3–5′ I 1–5′ R (x1-4); 200 mm Hg or 50 mm Hg> Systolic BP (Elective PCI)	Wide range	RIPC significantly reduced incidence of perioperative MI and CI AKI
Zografos et al., Metanalysis [44]	1,066	Upper/Lower limb 3–5′ I 3–5′ R (x1-3); 200 mm Hg (Elective PCI)	Wide range	RIPC reduces incidence of peri-procedural MI
D′Ascenzo et al. (Metanalysis) [41]	731	Upper/Lower limb 5′ I 5′ R (x3); 200 mm Hg (x2–3) (Elective PCI/PCI for unstable angina)	Wide range (Immediately before, up to 2 h before PCI)	Reduced incidence of perioperative MI (defined by increase of cTnT to >3x 99th percentile URL); more benefit when RIPC was delivered on lower extremity
Lavi et al. [36]	360	Upper limb vs. Lower limb 5′ I 5′ R (x3); 200 mm Hg (PCI for stable/unstable angina)	Post PCI	RiPostC did not change the incidence of peri-procedural MI
Carrasco-Chinchilla et al. [33]	232	Upper limb 5′ I 5′ R (x3); 200 mm Hg Post PCI (Elective PCI for stable/unstable angina)	Post PCI	RiPostC did not reduce troponin release, peri-operative MI and had no impact on clinical outcomes
Xu et al. [39]	200	Upper limb 5′ I 5′ R (x3); 200 mm Hg (Elective PCI); Included patients with DM & >/=65 years of age)	<2 h prior to PCI	No significant difference in release of hs-cTnI or incidence of post-procedure MI 4a
Liu et al. [37]	200	Upper limb 5′ I 5′ R (x3); 200 mm Hg (Elective PCI)	18 h before PCI	Reduced release of cardiac enzyme post-PCI and reduced adverse events at 6 months
CRISP stent trial Hoole et al. [31] Davies et al.[34]	242 in initial trial (192 in 6 years follow-up)	Upper limb 5′ I 5′ R (x3); 200 mm Hg (Elective PCI)	Variable time before PCI	Reduced perioperative myocardial injury; Reduced MACCE rate at 6 months and 6 years (HR 0.58 *p* = 0.039). RIPC protection was seen early (within 1–2 h of RIPC); No difference in MACCE at 6 years in diabetics
Ahmed et al. [32]	149	Upper limb 5′ I 5′ R (x3); 200 mm Hg (Elective PCI)	Variable time before PCI	Reduced procedure-related cTnT release
Prasad et al.[38]	95	Upper limb 3′ I 3′ R (x3); 200 mm Hg (Non-emergent PCI for stable/unstable coronary disease)	Immediately before PCI	No difference in post procedure cTnT release
Zografos et al. [40]	94	Upper limb 5′ I 5′ R (x1); 200 mm Hg (Elective PCI)	Immediately before PCI	Reduced peri-procedural cTnI release and lesser incidence of peri-operative MI
MERIT trial Ghaemian et al.[35]	80	Lower Limb 5′ I 5′ R (x2) (Elective PCI)	1 h before procedure	Reduced release of cTnT at 24 h
Remote Ischemic Conditioning in Acute Myocardial Infarction				
ERIC-LYSIS [16] Yellon et al.	519	Upper limb 5′ I 5′ R (x4); 200 mm Hg (Thrombolysis for STEMI)	RIPC before thrombolysis	RIPC reduced AUC for cTnI and CK-MB release post pPCI
Sloth et al. [14, 43]	333	Upper limb 5′ I 5′ R (x4); 200 mm Hg (pPPCI for STEMI)	Before PCI (in-ambulance)	Significant reduction in HR for MACCE extending beyond 3 years
RIPOST-MI Prunier et al. [13]	151	Upper limb 5′ I 5′ R (x3); 200 mm Hg (STEMI patients undergoing pPCI)	Perconditioning (Before PCI)	RIPerconditioning significantly reduced Peak CK-MB, CK-MB AUC to area at risk (AAR) ratio, and peak CK-MB level to AAR ratio
Crimi et al. [11]	100	Lower limb 5′ I 5′ R (x3); 200 mm Hg (Anterior STEMI patient undergoing pPCI)	post PCI	RiPostC reduced peri-operative myocardial infarction measured by AUC for CK-MB release, reduced T2-weighted edema on MRI
Yamanaka et al. [15]	94	Upper limb 5′ I 5′ R (x3); 200 mm Hg (STEMI patients undergoing pPCI)	With pPCI	Reduced incidence of contrast induced AKI
Metanalyses combining studies of Remote Ischemic Conditioning in both emergent and non-emergent setting				
Zuo et al. (Metanalysis) [45]	1542	Various protocols including RIPostC (various indications for PCI)	Variable (before/after PCI)	RIPC/RIPostC reduces the incidence of PCI related AKI; Also RIPC reduces in hospital mortality and MACEs
Yetgin et al. (Metanalysis) [43]	557 (PCI) 314 (pPCI) 891 (CABG)	Various protocols in the setting of PCI (pPCI for STEMI and CABG)	Variable	RIPC reduced perioperative myocardial necrosis, measured by increase in cardiac enzymes (overall analysis showed a trend but were not significant for PCI studies)

*DM* diabetes mellitus. *HR* hospitalization rate

RIPC has also been evaluated for its ability to protect the myocardium and improve clinical outcomes in patients presenting with an acute myocardial infarction (both NSTEMI and STEMI) who undergo PCI [11–16]. Since the myocardium is already ischaemic in these patients when they present with an evolving infarction, RIPC protocols in this setting are referred to as “per-conditioning” (RIPercon) when RIPC is given prior to PCI, and as “post-conditioning” (RIPostC) when given during or after PCI. Results are more promising in this setting and demonstrate that RIPercon or RIPostC can reduce myocardial injury and acute kidney injury related to PCI as well as improve long-term clinical outcomes in patients undergoing PCI for acute myocardial infarction, in terms of MACCE comprising of a composite of all-cause mortality, myocardial infarction, readmission for heart failure, and ischemic stroke/transient ischemic attack [14]. Interestingly, metanalyses of trials in patients undergoing both elective and emergent PCI (for acute myocardial infarction) have shown a benefit for RIPC in terms of both myocardial and kidney injury, although data from large-scale, randomized clinical trials is still missing [41–45]. Despite the more consistent benefit observed in this setting, a wide range of protocols have been utilized to evaluate the role of RIPC in PCI (Table 2) – which vary in the number of cycles of RIPC [1–4], the duration of cuff inflation/deflation (1–5 min), the cuff inflation pressure used (200 mmHg or 50 mmHg above resting blood pressure), choice of limbs(s) used for RIPC and the timing of RIPC with regards to the PCI or the ischemic insult (i.e. RIPercon or RIPostC). As with CABG, additional factors can potentially influence the effects of RIPC, including patients’ comorbidities, co-medications, as well as some of the agents typically used to reduce myocardial injury in the setting of acute MI such as heparin, antiplatelet agents, statins, and anti-coagulants - all of which may mask or minimize the potential additional benefit of RIPC.

Larger trials are currently underway including the ERIC-pPCI trial (evaluating RIPC in the setting of pPCI for STEMI) and the EURO-CRIPS trial [46] (evaluating RIPC in the setting of PCI being performed for all indications apart from STEMI), which will help to clarify if RIPC truly has a beneficial impact on clinical outcomes in patients undergoing PCI.

Yet another field in which RIPC may be effective, is in preventing perioperative ischaemic myocardial injury in heart transplantation surgery [47]. Konstantinov et al. showed that in pigs, RIPC of the recipient animal could reduce ischemia-reperfusion injury sustained by the donor heart after orthotopic transplantation [48]. In their study, four 5-min cycles of lower limb ischemia and reperfusion in the recipient animals served as the RIPC stimulus and the left anterior descending artery of the transplanted heart was occluded for 30 min following heart transplantation to test whether there was protection against ischemia and reperfusion injury [48]. Remotely preconditioned recipient pigs sustained myocardial infarctions in the transplanted hearts that were 63 % smaller than control pigs. Another interesting conclusion that may be made from this experiment is that neural innervation to the heart is not essential for delivery of the cardioprotective stimulus.

Interestingly, RIPC has also been shown to be effective in preventing myocardial injury even in the setting of non-cardiac surgery such as abdominal aortic aneurysm repair. Ali et al. determined that RIPC could reduce myocardial injury as well as renal injury in patients undergoing open abdominal aortic aneurysm repair [49]. RIPC was achieved using two cycles of occlusion of the common iliac artery with 10 min ischemia followed by 10 min reperfusion. RIPC reduced the incidence of myocardial injury and myocardial infarction by 27 and 22 % respectively (assessed by measurement of perioperative release of Troponin I [49]. The mechanism of myocardial injury in this setting is unclear.

Thus RIPC has shown a potential for being able to protect the heart against lethal ischemia reperfusion in a wide range of clinical settings, though proof of its utility in improving clinical outcomes in these settings from large randomised controlled clinical trials is still awaited. There are several hurdles in its clinical implementation, discussed above, which have so far prevented RIPC from being applied on a routine basis in clinical practice. This could change in the future as we understand better the limitations in its application as well the mechanisms that underlie this cardioprotective phenomenon.

### Mechanisms Underlying RIPC Mediated Protection of the Heart

#### Innate Protective Pathways within the Myocardium Activated by RIPC

The mechanisms within the heart that lead to cardioprotection from RIPC are similar to those involved in direct IPC of the heart [50]. IPC itself appears to begin with ligand binding to G-protein coupled receptors on the surface of the cardiomyocytes. These ligands include adenosine, bradykinin, opioids, angiotensin and endocannabinoids. The binding of cardiomyocyte surface receptors to the respective ligands initiates signalling pathways, which eventually lead to changes in the myocardium, rendering it resistant to ischaemia and reperfusion injury [50, 51]. One of the important innate cardioprotective pathways activated via IPC is the Reperfusion Injury Salvage Kinase (RISK) pathway [50]. Cardioprotection from RIPC has, similarly, been shown to be associated with phosphatidylinositol-3-OH kinase (PI3K)-Akt activation, which is an integral component of the RISK pathway [52], suggesting similar mechanisms underlying IPC and RIPC.

Activation of the RISK pathway is known to inhibit the mitochondrial permeability transition pore (mPTP), which appears to be the final target of this cardioprotective pathway [53]. Opening of the mitochondrial permeability transition pore (mPTP) during reperfusion results in both necrotic cell death, due to ATP depletion, and apoptotic cell death, due to swelling and rupture of mitochondria [54]. Though, it is clear how preventing the opening of mPTP by IPC provides cardioprotection, direct evidence linking RIPC to inhibition of mPTP opening has yet to be obtained. RIPC-induced protection from reperfusion injury has also been shown to be mediated by the activation of Signal Transducer and Activator of Transcription (STAT)-3 proteins which act through a different cardioprotective pathway, called the survivor activating factor enhancement (SAFE) pathway [55]. There are many proposed mechanisms to explain how the SAFE pathway confers protection to the myocytes [56], with evidence that the mPTP may again be the key end effector [57]. The RISK and SAFE pathways are not mutually exclusive and there is evidence of interaction between them [55].

There is much evidence to suggest that another pathway involving ATP-sensitive potassium (K_ATP_) channels play a role in cardioprotection offered by IPC. In particular mitochondrial K_ATP_ channels have been implicated in this protection, with Garlid et al. [58] and Liu et al. [59] demonstrating that K_ATP_ opener diazoxide mimicked the cardioprotective effects of IPC and was a far more potent opener of mitochondrial K_ATP_ than sarcolemmal K_ATP_. Similar to its role in mediating protective effects of IPC, K_ATP_ channels have been implicated in cardioprotective effects of RIPC, working independently of the neural pathways of RIPC protection [48]. There are several theories that attempt to explain how the opening of K_ATP_ channels could lead to cardioprotection. Costa et al. demonstrated that opening of K_ATP_ channels inhibits the opening of the mitochondrial permeability transition pore (mPTP), similar to the RISK and SAFE pathways, leading to cardioprotection [60]. Opening of K_ATP_ channels appears to lead to the activation of downstream protein kinases like Protein kinase C (PKC), via generation of reactive oxygen species during reperfusion. These downstream kinases (in particular the PKC-ε isoform) then relay the cardioprotective signal to the end-effectors of cardioprotection leading to the inhibition of the opening of mPTP [61]. Protein kinase C (PKC) has additionally been shown to play a critical role as a mediator of the preconditioning signal in IPC with many studies demonstrating the role of the PKC-ε isoform in cardioprotection from IPC [61–63]. Likewise, studies have linked PKC-ε activation with RIPC by showing that the cardioprotective effects of RIPC can be abolished by PKC blocker chelerythrine [64]. The intracellular targets of PKC have yet to be established, however.

#### Mechanisms Involved in Transmission of RIPC Signal to the Heart

**Fig. 1:**
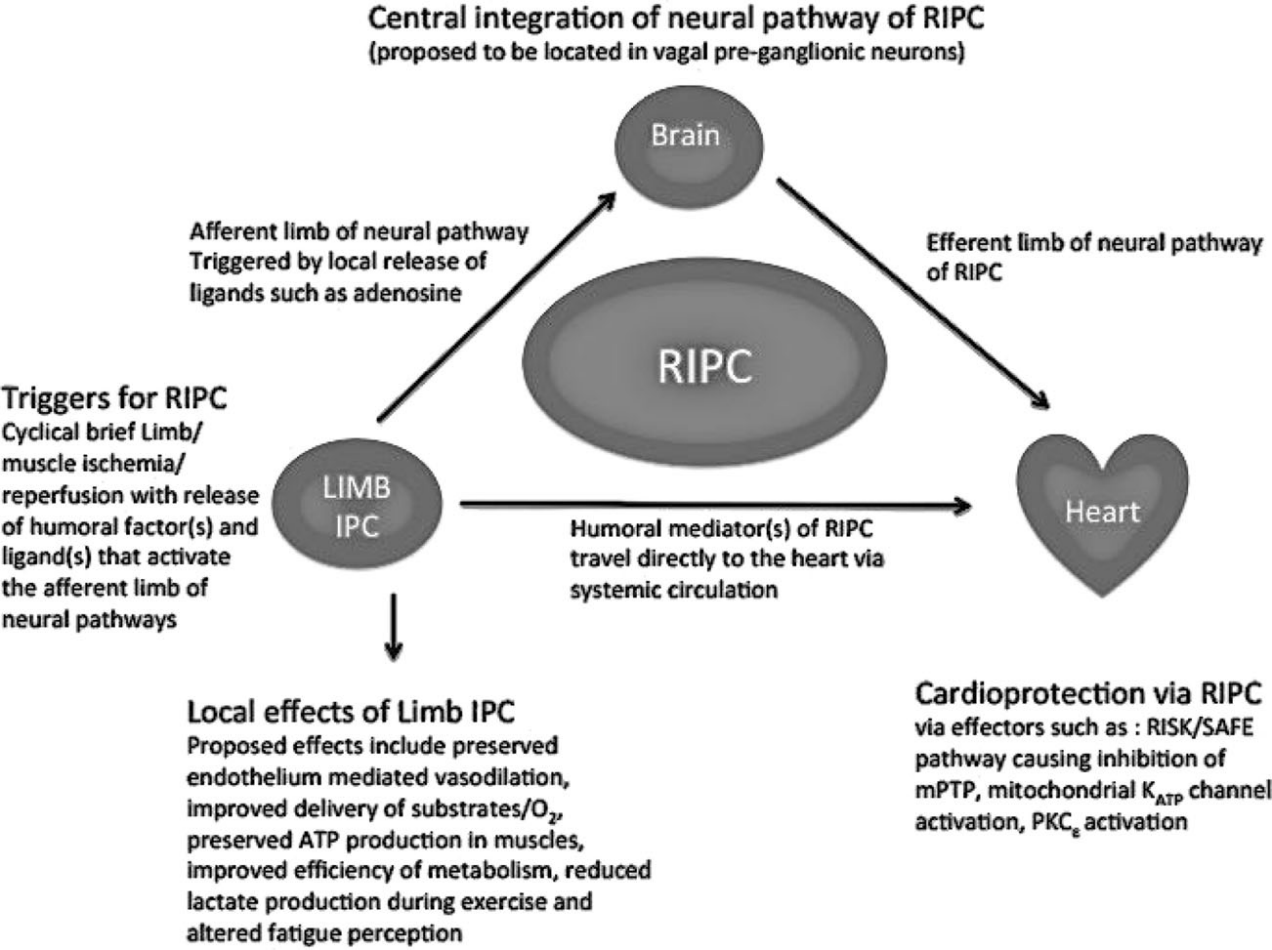
Schematic diagram of the mechanisms involved in the local and remote (cardioprotective) effects of limb IPC

Intriguingly, though the protective changes within the myocardium that take place secondary to RIPC are well studied (as reviewed above), the means by which the protective stimulus is communicated to the heart from the remote tissue on which RIPC is performed remains unclear. The two primary theories for mechanistic pathways linking the remote tissue to the heart involve either the release of a humoral factor in the remote tissue that is transported to the heart or a protective signal transmitted through a neural pathway between the remote tissue and the heart (Fig. 1) [65]. These pathways are not mutually exclusive and it is likely they interact with each other to facilitate RIPC. This idea is supported by a recent study, which found that the cardioprotective effect of RIPC was partially blocked by either femoral vein occlusion (humoral pathway) or sciatic nerve resection (neural pathway) but completely abolished by a combination of the two [65]. This also suggests that both mechanisms may (at least partially) transmit the cardioprotective signal independently to the heart.

##### Humoral Pathway of Transmission of RIPC Signal to the Heart

There have been many experimental studies, which support the idea that a humoral factor released at the remote tissue plays a role in RIPC. Studies have shown that rabbits which received either whole blood transfusion or coronary effluent transfusion from donor rabbits that had undergone ischaemic preconditioning are as well protected from myocardial infarction as donor preconditioned rabbits [66]. The fact that this protection was successfully transferred from preconditioned rabbits to untreated rabbits via blood transfusion supports the idea that a humoral factor conveys the preconditioning signal from the remote organ to the heart. This theory is also reinforced by the previously mentioned study by Konstantinov et al., which showed that RIPC of the recipient could reduce myocardial infarction sustained by the denervated donor heart. This implies that protection can be transferred through the blood, independent of the neural pathway [48].

Evidence that a humoral factor is involved in RIPC may be strong, but the identity of such factors is still under investigation. A study by Serejo et al. using coronary effluent transfusion from preconditioned mice to untreated mice demonstrated that the cardioprotective factor released by IPC is thermolabile, hydrophobic substance with a molecular weight greater than 3.5 kDa [67]. A subsequent study by Shimizu et al. demonstrated that the cardioprotective factor had a molecular weight less than 15 kDa [68]. A recent study from our own lab found that plasma levels of stromal cell-derived factor-1 (SDF-1α), a chemokine of ~10 kDa size, is significantly increased in rat plasma following RIPC, and that AMD3100, a highly specific inhibitor of SDF-1α receptor CXCR4, blocked the cardioprotection afforded by RIPC [69]. Intriguingly, this chemokine falls in the size range described for the humoral factor involved in RIPC in other studies cited earlier. Another humoral factor implicated in RIPC is Calcitonin gene-related peptide (CGRP) and this factor appears to work via activation of PKC-ε in the myocardium after a RIPC stimulus [70]. However, disappointingly, in an extensive human proteomic analysis by Helgeland et al., no plasma protein could be conclusively demonstrated to be regulated via RIPC, although they recognized that this method has limited sensitivity in the range of low to medium abundance proteins [71]. They suggested that further research should focus on smaller humoral factors, which may work in conjunction to transmit the protective signal of RIPC. Interestingly, we have recently shown that exosomes, which are cell-derived vesicles 30–100 nm in size, can transmit a cardioprotective signal via the blood [72]. Exosomes and similar extracellular vesicles carry a number of signalling agents, such as micro-RNAs (miRNAs), which play a regulatory role in a number of intracellular pathways and may have a role to play in RIPC, though this has yet not been established [73–75]. miRNAs themselves (in particular miRNA-144 and miRNA-1) have also been implicated in RIPC mediated protection [40, 76, 77].

A number of studies have demonstrated that the protection offered by RIPC to the heart and other organs can be abolished using inhibitors of various opioid and cannabinoid receptors [78–83]. This suggests that endogenous opioids and cannabinoids may be released at the remote organ during RIPC and carried to the heart in the blood where they act directly on receptors in the myocardium or other organs being targeted via RIPC, although it is also possible that they are released locally, in the heart. Furthermore, there is evidence for the involvement of angiotensin-1 receptors and hypoxia inducible factor-prolyl 4-hydoxylases in cardioprotection induced through RIPC via occlusion of the renal artery [84, 85].

In summary, despite a number of studies showing a role of various humoral factors in the transmission of the protective effects of RIPC from a remote organ to the heart, no single factor has to date been conclusively identified as the mediator of this protection. Identification of such as factor would greatly aid clinical implementation of RIPC, as it would enable optimization of RIPC protocols and optimal timing for this intervention to achieve the maximum cardioprotection by RIPC.

##### Neural Pathway of Transmission of RIPC Signal to the Heart

While numerous studies that have explored the humoral pathways of RIPC protection, others have likewise shown a crucial role of a neural pathway of protection underlying RIPC [86, 87]. In one of the first studies of RIPC, Gho et al. showed that ganglion blockade by hexamethonium eliminated the cardioprotection induced by RIPC [6]. A study by Liem et al. showed that RIPC led to local release of adenosine that activated a neural pathway, which subsequently leads to activation of myocardial adenosine receptors, implicating a role for adenosine in the neural pathway of RIPC protection [88]. A study by Ding et al. also demonstrated that adenosine released in the remote organ undergoing ischemia-reperfusion, to induce RIPC, stimulates afferent nerve signals that confer cardioprotection [89], similar to the study by Liem et al.

Interestingly, the first evidence for the involvement of adenosine in RIPC was obtained in 1998, when Pell et al. demonstrated that the administration of the non-selective adenosine receptor antagonist 8-sulfophenyltheophylline before RIPC at the renal artery abolished its cardioprotective effects [90]. This finding was supported by Takaoka et al., who found that 8-sulfophenyltheophylline administration after the RIPC protocol had the same effect. They also discovered that plasma levels of adenosine in carotid artery blood were higher in rabbits subjected to RIPC (delivered via renal artery occlusion), compared to rabbits subjected to IPC within the heart itself, providing further evidence that adenosine is involved in signalling between the remote tissue and heart in RIPC [91]. Evidence that adenosine plays an important role in the neural pathway of RIPC protection was later provided in the aforementioned studies by Liem et al. [88] and Ding et al.[89]. Dong et al. provided more evidence supporting a neural pathway mediated by adenosine in RIPC by demonstrating that intrafemoral artery injection of adenosine produced similar cardioprotective effects to those of RIPC delivered via femoral artery occlusion, and that femoral nerve section abolished the effects of the RIPC [92]. Much of the experimental evidence therefore suggests that RIPC generates adenosine in the remote tissue, which then activates afferent sensory nerves. However, it is also possible that adenosine also acts as a humoral factor, binding directly to receptors within the myocardium. There is some evidence showing that other signalling molecules such as bradykinin may also play a role in the neural pathway of RIPC induced protection [64, 93]. Mastitskaya et al. have demonstrated a crucial role of preganglionic vagal neurones located in the brainstem dorsal motor nucleus of the vagus nerve in the cardioprotection offered by RIPC, suggesting the neural pathway may be centrally integrated and regulated [87].

In conclusion, numerous triggers, mediators and effectors of RIPC have been investigated leading to considerable progress towards our understanding how the effects of RIPC on the heart may be mediated. The current evidence seems to support the idea that many pathways and physiological systems are affected acutely by RIPC, suggesting that cardioprotection may not be the only outcome of using this intervention.

## Improving Exercise Performance with Limb IPC

The demonstration of the favourable effects of IPC and RIPC on endothelial function, skeletal muscle and the heart raised the possibility that, in addition to being of benefit in a clinical setting, limb IPC could also be applicable in sport. Intense exercise has been shown to lead to cardiac and skeletal muscle hypoxemia and may therefore represent a form of ischaemic insult [94]. A number of recent studies have investigated whether IPC applied to the limb prior to exercise can improve exercise performance (Table 3), via local effects on the limb/s and/or via mechanisms acting remotely on other organs, particularly the heart. The evidence for limb IPC improving exercise performance and the proposed mechanisms behind it will be examined in this section.

**Table 3:** Summary of trials exploring the benefits of limb IPC in improving exercise performance (I = ischemia, R = reperfusion)

Study	Exercise Setting/Fitness level of subjects	No. of subjects	Limb IPC stimulus: limb(s) used, duration of I-R (min), number of cycles, cuff inflation pressure	Timing of Limb IPC/RIPC	Time between exercise session/Sham inflation pressure	Results
Tocco et al. [108]	5000 m self-paced running /Skilled runners (10–12 h/week of training)	11	Both lower limbs 5′ I 5′ R (x3); 50 mmHg above resting systolic	5 min before exercise (no warm-up)	7–14 days, 10 mm below diastolic pressure	No change in average running speed, oxygen uptake, aerobic energy cost during exercise and post-race blood lactate
Patterson et al. [109]	Repeated sprint test during cycling ergometry/ Mean 6.7 +/− 2.3 h per week of training	14	Both lower limbs 5′ I 5′ R (x4); 220 mmHg	30 min before warm up for exercise	5–7 days, 20 mmHg	Limb IPC improved peak and mean power output during the early stages of repeated sprint cycling
Gibson et al. [107]	Maximal sprinting performance over 30 m/ Well trained individuals	25	One lower limb 5′ I 5′ R (x3); 220 mmHg	5 min before warm up	<7 days, 50 mmHg	No improvement in sprint speed with limb IPC
Bailey et al. [99]	Graded maximal treadmill running test, followed by a 5-km time trial /Moderately trained individuals <10 h/week	12	Both lower limbs 5′ I 5′ R (x4); 50 mmHg above resting systolic	20 min before running test and 100 min before 5-km time trial	5–7 days, 20 mmHg	Lower Limb IPC preserved post exercise brachial artery endothelium-dependent function (remote effect)
Bailey et al. [103]	5 km time trial/Moderately trained individuals	13	Both lower limbs 5′ I 5′ R (x4); 220 mmHg	90 min before 5 km time trial	Not available, 20 mmHg	IPC improved 5-km time trial performance; IPC was associated with lesser rise in blood lactate concentration at submaximal level during incremental running test.
Crisafulli et al. [102]	Incremental, maximal exercise tests on a cycle ergometer/Healthy individuals	17	Both lower limbs 5′ I 5′ R (x3); 50 mmHg above resting systolic	5 min before exercise	>7 days, No sham group	Limb IPC improved maximal exercise performance but not maximal oxygen uptake
Jean-St-Michel [104]	Swimming/Competitive swimmers (with previous times meeting Canadian national championship qualification standards)	16–18	Upper limb 5′ I 5′ R (x4); 15 mmHg above resting systolic blood pressure	Immediately before warm-up	>7 days, 10 mmHg	Improved maximum swim time. Dialysed blood taken from subjects after RIPC reduced myocardial infarct in mouse Langendorff heart preparations
de Groot et al. [95]	Incremental maximal exercise tests on a bicycle ergometer (Well trained cyclists)	15	Both lower limbs 5′ I 5′ R (x3); 220 mmHg	5 min before exercise	>7 days apart, No sham group	IPC had no effect on ventilation, respiratory quotient, maximal heart rate, blood pressure or blood lactate. Limb IPC improved maximal oxygen consumption

In 2009, De Groot et al. hypothesised that limb IPC would improve exercise performance and maximal oxygen consumption [95]. In their study of well-trained cyclists they found that preconditioning by three 5 min cycles of ischemia and reperfusion in both legs using blood pressure cuffs inflated to 220 mmHg significantly increased maximal oxygen uptake and power output in healthy subjects exercising on a bicycle ergometers [95]. The authors speculated that enhancement of skeletal muscle vasodilation could have explained the improvements in performance. Limb IPC may achieve these effects through the activation of vascular smooth muscle K_ATP_ channels and local release of adenosine, which can both contribute to vasodilation in muscles [96, 97] during exercise. Both the activation of K_ATP_ channels and the local release of adenosine have also been implicated as mediators of the effects of RIPC, as previously outlined. Previous studies have shown that limb IPC can additionally protect the endothelium from ischemia-reperfusion injury both locally and remotely, preserving endothelial function in the wake of ischemia [10, 98] and these effects might improve exercise performance by maintaining the supply of oxygen and energy substrates to the skeletal muscles during intense exercise, when the exercising muscle groups might be subject to hypoxia, thereby sustaining the contractile activity. This possibility is further supported by a study which showed that an IPC stimulus applied to both legs prior to a 5 km running time trial was able to prevent the decrease in brachial artery endothelial function that would occur otherwise with strenuous lower extremity exercise, suggesting a remote effect of limb IPC in preserving blood flow remotely during exercise [99]. Limb IPC also appears to have an positive influence on the coronary circulation during exercise through an increase in coronary blood flow and a reduction in the coronary vascular resistance [100, 101], though the exact mechanism for this change in coronary flow is not known currently and its impact on the exercise capacity may be somewhat limited However, consensus on the positive effects of RIPC on exercise performance is still lacking.

In fact, a recent study by Crisafulli et al. exploring the effects of limb IPC on incremental exercise performance on a cycle ergometer, exhibited no change in oxygen uptake, stroke volume or cardiac output during exercise. However, they noted an increase in maximal exercise performance after limb IPC [102]. They postulated that the beneficial effect of limb IPC could be related to an alteration in the perception of fatigue. In our own study using healthy volunteers, we have shown that the perception of pain/discomfort in relation to cuff occlusion for RIPC reduces with subsequent cuff inflations [28], possibly either due to some desensitisation of nerve signal at the site of occlusion with repeated exposure or due to central alteration in pain perception. Although, it may be postulated that limb IPC may beneficially modify fatigue/pain perception during subsequent exercise, the mechanisms and whether or not they are systemic or locally mediated remains to be elucidated.

Other studies showing beneficial effects of limb IPC on exercise performance include a study by Bailey et al. which showed that IPC (involving 5 min BP cuff inflation at 220 mmHg over both lower extremities) improved 5 km time trial performance in healthy males and also reduced lactate accumulation at sub-maximal level during an incremental running test [103]. The mechanism by which blood lactate accumulation during exercise is attenuated via limb IPC is unknown. Bailey et al. speculated that reduced ATP consumption or increased efficiency of excitation-contraction coupling during exercise caused by the preceding IPC, could have led to the reduction in muscle lactate production. Interestingly, a study in pigs has shown that IPC of skeletal muscles can preserve the muscle ATP level at the end of lethal ischemia-reperfusion as well as lower the accumulation of lactate, providing some support to this presumption [2].

Additional evidence that limb IPC has the potential to improve maximal performance comes from Jean-St-Michel et al. who found that limb IPC improved maximal performance in national level swimmers [104]. They demonstrated that IPC involving 4 cycles of upper limb ischemia for 5 min (induced by BP cuff inflation to 15 mmHg above resting systolic BP) each followed by 5 min of reperfusion, improved 100 m swim time by an average of 0.7 s, which is of major competitive significance over such a race distance [105]. This study supports the idea that limb IPC improves maximal performance through remote actions, as the IPC stimulus was administered only in one limb, but swimming itself involves several muscle groups and all extremities. This is reinforced by the strong clinical evidence that the benefits of RIPC are mediated by both the systemic release of a humoral factor(s) as well as the activation of neural pathways that can affect a number of remote organ systems. In the latter study, authors speculated that increased ATP production might contribute to the increase in performance. There is certainly some evidence that IPC of the myocardium can preserve ATP production during subsequent lethal ischemia via its effects on mitochondrial K_ATP_ channels [106], but whether limb IPC has similar beneficial effects on ATP production locally in skeletal muscles or remotely, remains to be shown. While some positive effects of limb IPC seem possible in endurance activities and swimming, it seems that IPC does not consistently improve sprinting performance. In fact, recent work from Gibson et al. showed that an IPC protocol of 3 cycles of 5 min occlusion applied unilaterally (3 × 5 min on each leg) at 220 mmHg had no effect on maximal sprints over 30 m [107]. Furthermore, IPC does not seem to improve performance during self-paced 5 km running on an outdoor track or acutely affect oxygen uptake and aerobic energy cost during the race [108]. The post-race lactate levels were also unaffected by limb IPC in this study conducted in well trained men [108]. These results are completely opposite to the findings of Bailey et al. (2012) which showed an improvement in 5 km time trial on a treadmill following an incremental test in healthy (but not well trained) individuals [103], suggesting that maybe the beneficial effects can only be evident in untrained populations or perhaps that prior training can mimic the benefits of limb IPC, such as by chronic limb ischemic preconditioning by exercise induced hypoxia.

However, while maximal sprints may not benefit from IPC, repeated sprints performed on a cycle ergometer seem to offer a difference perspective. Patteron and coworkers recently showed some marked beneficial effects of limb IPC (4 × 5 min cuff inflation on both extremities at 220 mmHg followed by 5 min reperfusion) on repeated sprint cycling performance as well as improvement in peak and mean power output over the initial sprints [109]. The study also showed that IPC maintained a better tissue saturation index during exercise, compared to placebo, suggesting that improved oxygen delivery to the muscles could be a contributing factor. Interestingly, in this study there was no difference in fatigue perception between IPC and placebo, although it should be noted that subjects’ warm up prior to exercise was carried out 30 min after IPC delivery which is in contrast to other studies where warm up and exercise began soon after IPC. A clear area of potential future investigation therefore relates to timing of IPC delivery in further understanding its potential beneficial effects. Clearly more studies are needed to evaluate which aspect of sprint performance are positively influenced by limb IPC as well as to develop a clearer understanding of the underlying mechanisms.

It is known that group III and group IV afferent sensory neurons respond to mechanical and metabolic alterations in the skeletal muscles during exercise and initiate a reflex that is transmitted to the cardiovascular regulation centers in the brain stem, which in turn increases sympathetic efferent activity and reduces parasympathetic activity during exercise [110]. This cause an increase in the heart rate and cardiac output as well as vasoconstriction in non-active skeletal muscle vascular beds to divert blood flow to the actively exercising skeletal muscles. This effect on the sympathetic and parasympathetic outflow during exercise is necessary to meet the increase metabolic demands of the muscles being used actively during exercise. It can be postulated that a similar reflex pathway(s) may be activated by metabolic changes in the skeletal muscles during limb IPC, which may have a beneficial systemic and/or local effect during subsequent exercise or that metabolic changes in the skeletal muscles during limb IPC may somehow positively influence these neural pathways to improve exercise performance following IPC, through its effect on blood flow locally or systemically. However, so far the role of these neural reflexes in limb IPC has never been evaluated. Intriguingly, it appears that limb IPC is able to at least modulate local blood flow in the limb through increase in functional sympatholysis, which is a term given to a reduction in vasoconstriction that usually occurs during exercise due to sympathetic activation. This effect of limb IPC may occur via the activation of K_ATP_ channels in the vascular smooth muscles locally and increased local production of nitric oxide due to limb IPC [111]. It is important to recognize some key differences in the various experimental protocols used for both IPC and sham studies as well as the form of exercise used in these studies to investigate the effect of limb IPC as listed in Table 3 [95, 99, 102–104, 107–109]. The majority of studies have used either 220 mmHg or a fixed value (15 or 50 mmHg) above resting systolic BP. Based on the resting systolic pressure, there may have been a wide difference in the cuff inflation pressure used in these protocols. Similarly, while some studies listed in Table 3 did not use a sham group, others used a cuff inflation pressure ranging from 10 to 50 mmHg or 10 mmHg below resting diastolic blood pressure. Inflation of a blood pressure cuff can affect the arteriovenous pressure gradient and thereby limb perfusion [112, 113]. While this effect may be negligible at very low pressures such as 10–20 mmHg, the effect of slightly higher pressures such as 50 mmHg or 10 mmHg below the diastolic blood pressure is unknown, and could potentially influence performance within the sham group. The use of a low cuff inflation pressure in the sham group also makes it difficult to adequately blind subjects to their treatment, and could subconsciously influence the performance of participants. Other variables in these studies include the inclusion or absence of a warm up period between limb IPC and exercise, and the time interval between experimental sessions (which ranged from 5–7 days to 7–14 days). IPC and RIPC result in a “second window” of protection that lasts 24–72 h [98, 114], but the time course of beneficial effects of limb IPC in the exercise domain has not been established. It seems reasonable to assume that improvements to performance are unlikely to last beyond 1 week. The tested exercise protocols themselves vary widely and involve different muscle groups (running, swimming, cycling) and the physiological changes in the body during these different forms of exercise may also vary significantly, thereby having an impact on the results. Finally, the subjects in these studies range from “healthy individuals” to “well-trained athletes”, some of whom compete at a national level. Although such individuals are likely to vary markedly in their response to RIPC, these differences have not yet been determined. It is important to take such factors into account, not only in interpreting the results of previous trials, but also in the design of future studies.

In conclusion, the effect of limb IPC in sports performance is a relatively unexplored area of study and despite some evidence suggesting its benefits in this domain, there is currently insufficient experimental proof to draw confident conclusions about its potential to acutely enhance performance with particular reference to elite athletes. The mechanisms underlying this potential benefit are similarly very poorly understood and under-investigated. Finally, it is unclear whether limb IPC can add significant value over and above a well-planned warm-up, particularly in well-trained, elite athletes [115–119]. In addition, application of limb IPC or RIPC may pose an ethical dilemma regarding the appropriateness of its use to improve exercise performance. This is particularly relevant now, as controversies about the use of performance enhancing drugs mar several sports.

## Future Considerations

Clearly, there is need for further research, particularly with regards to the mechanisms underlying the local and systemic effects of limb IPC in order to clearly establish how and if they can provide clinically significant cardioprotection in various settings discussed above and/or improve exercise performance. Currently there has been little more than speculation about the mechanisms, and the possibility that limb IPC improves performance via neural mechanisms has not been examined. Furthermore, it is unclear whether the upper or lower limb is more effective in inducing the positive effects of limb IPC/RIPC in terms of both potency as well as tolerability also in the clinical setting. While tolerability and discomfort associated with the limb IPC/RIPC protocol is not an issue in anaesthetized subjects undergoing CABG, this could be an important consideration for its wide spread utilization in awake subjects, both patients and athletes. We have recently shown that ischemia for RIPC can be more easily induced at lower pressures in the upper limb and that RIPC is better tolerated in the upper limb [28]. However, it is unclear whether the benefits of RIPC can be more effectively delivered through IPC applied to the upper or lower limb or both. In terms of RIPC delivery, there is currently no standardisation in methodology such as duration of cuff inflation, number of cycles/limbs(s) and the optimum cuff inflation pressure. It is unclear, whether or not limb circumference and/or subcutaneous fat mass affect duration and pressure of inflation that needs to be applied for RIPC. Clinical studies currently vary markedly in the number of cycles of RIPC used [2–4], cuff inflation pressure used for RIPC (200 mmHg vs. 15 mmHg above resting systolic pressure vs. 40 mmHg above resting systolic blood pressure), the number of limbs used for RIPC as well as the choice of limbs (upper, lower or a combination of the two) employed to deliver this cardioprotective intervention and the timing of RIPC in relation to the CABG (Table 1). Studies in the exercise domain have tended to use a higher cuff inflation pressure (220 mmHg) compared with clinical studies, which typically use 200 mmHg cuff inflation pressure for RIPC, though the rationale for this is unclear. Furthermore, criteria to aid practitioners in determining optimal dose–response are currently unknown. Clinical application of RIPC/limb IPC is also made more difficult by a lack of information about the optimal timing when RIPC should be delivered in relation to an ischemic insult/reperfusion and there are numerous factors in a real world scenario that can influence the delivery of this intervention in a timely fashion. Absence of a unequivocally proven clinical biomarker that can confirm the adequacy of the RIPC stimulus, considering the wide variation in protocols, as well as the optimal timing of its delivery, prevent clinical translation of this cardioprotective phenomenon. By analogy with direct IPC, there may be expected to be a first window of protection lasting 1–2 h, followed by a second window lasting 24–72 h [98, 114], but this remains to be investigated, to help define also the best timing for this intervention to be applied. It is probable that the ideal protocol for RIPC and limb IPC as well as their timing will vary based on the intended clinical/exercise setting for its application and the desired effects. However, dose–response studies to determine the optimal RIPC protocols for its different applications and whether or not certain subjects (such as patients with diabetes) respond in a different manner to a given RIPC stimulus than others, do need to be performed to clarify this issue.

## Conclusion

There is growing evidence that limb IPC is a promising non-invasive tool for protecting the heart, through its remote effects in the form of RIPC, and for improving some aspects of exercise performance, through both its local and remote effects. Clinical studies have so far been unable to show a consistent reduction in myocardial injury/infarction or improvement in clinical outcomes in the setting of coronary artery bypass grafting and PCI performed for various indications. Recently concluded large clinical trials, such as the ERICCA trial, that investigated the effect of RIPC on clinical outcomes in the setting of CABG have disappointingly been neutral. This highlights that we are far from widespread clinical application of this cardioprotective intervention. Further large clinical trials exploring the effect of RIPC on clinical outcomes in patients undergoing PCI are still underway and will answer if the current protocols being used to deliver this cardioprotective intervention can be beneficial in that scenario. A clearer understanding of the mechanisms underlying RIPC, identification of the humoral factor responsible for the transmission of the cardioprotective RIPC signal, investigation of the optimal protocols to be used for RIPC/limb IPC as well as delineating the time course of the effects are all needed to allow for the translation of the benefits of this non-invasive intervention into clinical practice as well as in the exercise domain. Following further animal and clinical studies, RIPC/limb IPC could potentially become a valuable technique both in clinical practice and for its applications in the exercise domain.

## Abbreviations

AAR: Area at risk
AKI: Acute kidney injury
AKT: Term used for protein kinase B
AT 1 receptor: Angiotensin II receptor type 1
ATP: Adenosine triphosphate
AUC: Area under the curve
BP: Blood pressure
CABG: Coronary artery bypass grafting
CGRP: Calcitonin gene-related peptide
CK-MB: Creatine kinase-myocardial band
CRISP stent: Cardiac remote ischemic preconditioning in coronary stenting
cTnI: Cardiac troponin I
cTnT: Cardiac troponin I
CXCR4: Chemokine receptor type 4
ERICCA: Effect of remote ischaemic preconditioning on clinical outcomes in patients undergoing coronary artery bypass graft surgery
ERIC-LYSIS: Effect of remote ischemic conditioning in heart attack patients
hs-cTnI: High-sensitivity cardiac troponin I
IPC: Ischemic preconditioning
IR: Ischemia and reperfusion
K_ATP_ channels: ATP-sensitive potassium channels
kDa: Kilodaltons
MACE: Major adverse cardiac events
MACCE: Major adverse cardiac and cerebrovascular event
MERIT: Myocardial event reduction with ischemic preconditioning therapy
MI: Myocardial infarction
miRNA: micro- ribonucleic acid
mPTP: Mitochondrial permeability transition pore
MRI: Magnetic resonance imaging
NSTEMI: Non-ST elevation myocardial infarction
PCI: Percutaneous coronary intervention
PI3K: Phosphatidylinositol-3-OH kinase
PKC: Protein kinase C
pPCI: Primary percutaneous coronary intervention
RIPC: Remote ischemic preconditioning
RIPercon: Remote ischemic perconditioning
RIPostC: Remote ischemic post conditioning
RIPOST-MI: Remote ischemic post-conditioning in myocardial infarction
RISK: Reperfusion injury salvage kinase
SAFE: Survivor activating factor enhancement
SDF = 1 α: Stromal cell-derived factor 1
STAT: Signal transducer and activator of transcription
STEMI: ST elevation myocardial infarction
